# Common Pathophysiology in Cancer, Atrial Fibrillation, Atherosclerosis, and Thrombosis: *JACC: CardioOncology* State-of-the-Art Review

**DOI:** 10.1016/j.jaccao.2021.08.011

**Published:** 2021-11-16

**Authors:** Orly Leiva, Duaa AbdelHameid, Jean M. Connors, Christopher P. Cannon, Deepak L. Bhatt

**Affiliations:** a Department of Medicine, Brigham and Women’s Hospital and Harvard Medical School, Boston, Massachusetts, USA; b Division of Hematology, Brigham and Women’s Hospital and Harvard Medical School, Boston, Massachusetts, USA; c Brigham and Women’s Hospital Heart & Vascular Center and Harvard Medical School, Boston, Massachusetts, USA

**Keywords:** arrhythmia, risk factor, thrombosis, AF, atrial fibrillation, CAD, coronary artery disease, CHIP, clonal hematopoiesis of indeterminate potential, CI, confidence interval, CLEC-2, C-type lectin-like receptor 2, HR, hazard ratio, IL, interleukin, MI, myocardial infarction, PCI, percutaneous coronary intervention, ROS, reactive oxygen species, TKI, tyrosine kinase inhibitor, VTE, venous thromboembolism

## Abstract

Cardiovascular disease and cancer are the 2 leading causes of death worldwide. Emerging evidence suggests common mechanisms between cancer and cardiovascular disease, including atrial fibrillation and atherosclerosis. With advances in cancer therapies, screening, and diagnostics, cancer-specific survival and outcomes have improved. This increase in survival has led to the coincidence of cardiovascular disease, including atrial fibrillation and atherosclerosis, as patients with cancer live longer. Additionally, cancer and cardiovascular disease share several risk factors and underlying pathophysiologic mechanisms, including inflammation, cancer-related factors including treatment effects, and alterations in platelet function. Patients with cancer are at increased risk for bleeding and thrombosis compared with the general population. Although optimal antithrombotic therapy, including agent choice and duration, has been extensively studied in the general population, this area remains understudied in patients with cancer despite their altered thrombotic and bleeding risk. Future investigation, including incorporation of cancer-specific characteristics to traditional thrombotic and bleeding risk scores, clinical trials in the cancer population, and the development of novel antithrombotic and anti-inflammatory strategies on the basis of shared pathophysiologic mechanisms, is warranted to improve outcomes in this patient population.

## Highlights

• Cancer and cardiovascular disease increasingly coexist, and patients with cancer are often undertreated.
• Cancer and cardiovascular disease share common pathophysiology, including inflammation.
• Thrombosis and bleeding risk scores often underperform in patients with cancer and cardiovascular disease.
• Inclusion of cancer status in cardiovascular trials and risk scores may improve cardiovascular outcomes.

Cardiovascular disease and cancer are the leading causes of death in the developed world (1). Advances in cancer therapeutics and diagnostics have led to improved survival and cancer-specific outcomes (2,3). As patients with cancer live longer, their risk for atherosclerosis, atrial fibrillation (AF), and cardiovascular disease likewise increases. Additionally, recent evidence suggests common pathophysiological linkages between cancer and cardiovascular disease. One study demonstrated an association between 10-year atherosclerotic risk score and the risk for incident cancer (4). In part, this is due to several shared risk factors, such as smoking, obesity, and diabetes mellitus. As new data emerge, the lines between cardiology, oncology, and cardio-oncology continue to blur (5).

The management of coronary artery disease (CAD) and AF often involve balancing thrombotic and bleeding risks associated with antiplatelet and anticoagulation therapy. Cancer-specific and therapy-related risk factors have been associated with an increased risk for thrombosis and adverse cardiovascular events in this patient population (6, 7, 8). However, patients with cancer also have an increased risk for bleeding compared with those without cancer. Therefore, management of CAD and AF in the cancer population adds an extra layer of nuance and complexity not seen in the general population. Numerous trials have been conducted attempting to find the optimal type and duration of therapy in a variety of prothrombotic cardiovascular conditions, though despite their increased thrombotic and bleeding risk, patients with cancer are often excluded from these trials (9, 10, 11). Thus, there is a relative paucity of evidence to guide antithrombotic therapy in patients with cancer and cardiovascular disease but an ample amount of opportunity to explore these nuances.

## An Inflammatory Common Link Among Atherosclerosis, AF, and Cancer

Recent evidence suggests that inflammation is the mutual pathophysiologic link among atherothrombosis, AF, and cancer (12,13). Additionally, atherosclerosis and cancer may influence the progression of each other. Elevated inflammatory markers, including high-sensitivity C-reactive protein and interleukin (IL)–6, have been shown to be a risk factor in the development of atherothrombosis and AF (14, 15, 16, 17). In AF, elevated C-reactive protein levels before ablation have been associated with increased likelihood of recurrent AF postablation (18). A causal role for inflammation in AF has been suggested in studies showing increased NLRP3 inflammasome activation in AF (19). Additionally, inflammatory markers have been shown to be reduced after successful ablation of AF (20).

Elevations in C-reactive protein and IL-6 have been associated with increased risk for cardiovascular events independent of cholesterol level (16,17,21). Additionally, NLRP3 inflammasome activation and IL-1β have been shown to promote atherogenesis and arterial thrombosis in preclinical animal models (22,23). This connection between inflammation and cardiovascular events has spurred trials investigating anti-inflammatory therapies in cardiovascular disease. In patients with rheumatoid arthritis, a population known to have high levels of inflammation and at elevated risk for cancer and cardiovascular disease, the ORAL Surveillance (Safety Study of Tofacitinib Versus Tumor Necrosis Factor [TNF] Inhibitor in Subjects With Rheumatoid Arthritis) study is examining the risk for major adverse cardiovascular events in patients with cancer receiving tofacitinib, a JAK1/3 inhibitor, compared with a tumor necrosis factor inhibitor (NCT02092467) (24,25). However, in the general population, anti-inflammatory therapies have shown promise in reducing cardiovascular events and cancer risk. The CANTOS (Canakinumab Anti-Inflammatory Thrombosis Outcomes Studies) trial randomized more than 10,000 patients with previous myocardial infarction (MI) and high-sensitivity C-reactive protein levels of 2 mg/L or higher to receive the IL-1β inhibitor canakinumab (50, 150, or 300 mg every 3 months) or placebo. The results of CANTOS showed significant reductions in nonfatal MI, nonfatal stroke, and cardiovascular death in patients treated with canakinumab (26,27). Colchicine is another anti-inflammatory drug studied in patients with chronic CAD and in post-MI populations (28,29). In patients with chronic CAD, low-dose colchicine significantly reduced MI and ischemia-driven revascularization compared with placebo (28). Additionally, there was a reduction in new onset or first recurrence of AF, though it did not reach statistical significance (28). In patients with recent MI, treatment with colchicine resulted in a significant reduction in the composite primary endpoint of cardiovascular death, resuscitated cardiac arrest, MI, stroke, or urgent hospitalization for angina leading to revascularization, with reductions in the latter 2 endpoints driving the observed effect (29).

**Figure 1:**
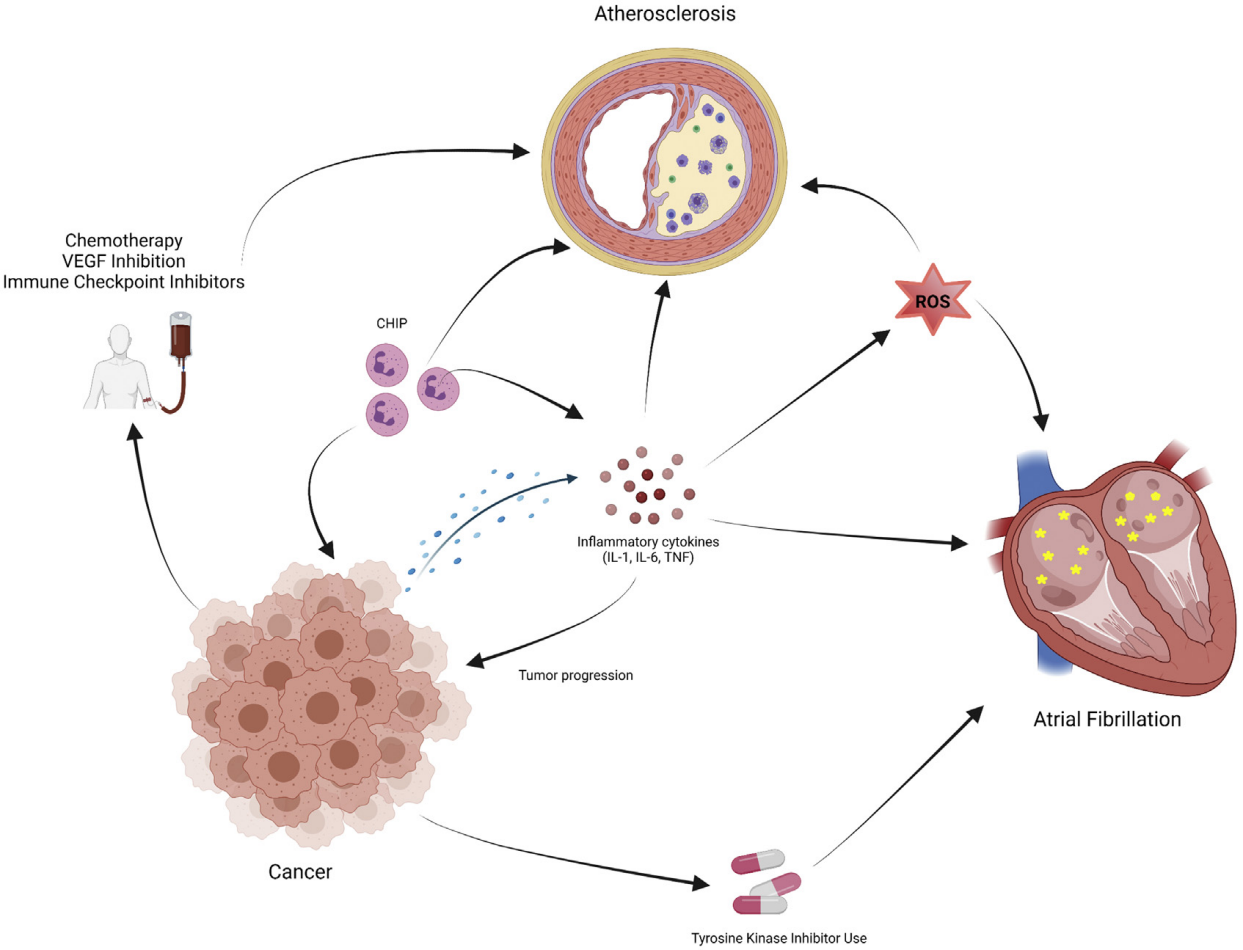
Inflammation and Cancer Treatment in Atrial Fibrillation and Atherosclerosis **Arrows** indicate relationships: inflammation arising from malignancy or other source as well as cancer-related treatment and clonal hematopoiesis of indeterminate potential (CHIP) can lead to progression of atherosclerosis and development of atrial fibrillation. IL = interleukin; ROS = reactive oxygen species; TNF = tumor necrosis factor; VEGF = vascular endothelial growth factor.

Inflammation also plays a role in the development and progression of cancer (30,31). IL-1 has been shown to mediate tumor angiogenesis, metastasis, and tumor immune evasion (32,33). IL-1 initiates and amplifies inflammation and can be produced by both immune (myeloid cells, macrophages, etc) and nonimmune cells in response to danger-associated molecular patterns and pathogen-associated molecular patterns (34). Additionally, IL-1 can induce the release of other proinflammatory and carcinogenic cytokines and factors, including IL-6, reactive oxygen species (ROS), and vascular endothelial growth factor (35,36). IL-6 and IL-1 cause activation of STAT3, which can induce proliferation of cancer cells and migration via increased expression of matrix metalloproteinases (37). Additionally, epithelial-to-mesenchymal transition, important in cancer cell invasiveness, metastatic potential, and cell detachment, is facilitated via IL-1, IL-6, and STAT3 signaling in chronic inflammation (37,38). The importance of IL-1 and IL-6 in the progression of cancer is highlighted in clinical trials of IL-1 and IL-6 antagonists on cancer survival. In an analysis of CANTOS, patients receiving canakinumab had a lower incidence of new lung cancer diagnosis and lower lung cancer mortality compared with those who received placebo (30). In 2 small trials (n = 47) in patients with smoldering multiple myeloma, the addition of the IL-1 receptor antagonist anakinra to dexamethasone was associated with improved progression-free and overall survival (39,40). Blockade of IL-6 with siltuximab inhibited progression of cholangiocarcinoma in a mouse model (41). Additionally, one retrospective study suggested lower incidences of cancer in patients with gout treated with colchicine, though it is unclear if this result is attributed to colchicine’s anti-inflammatory action or microtubule formation inhibition (42). The role of inflammation in the pathogenesis of cardiovascular disease and malignancy is an evolving field but one that is a common thread among these disorders (Figure 1).

Inflammation may lead to the development of AF, atherosclerosis, and cancer via the production of ROS. ROS are by-products of cellular metabolism and oxygen use and have been associated with increased risk for the development of cancer via DNA damage and genetic destabilization (43,44). In AF, production of ROS via leukocyte-derived myeloperoxidase may lead to atrial fibrosis and adverse extracellular matrix remodeling via matrix metalloproteinases (45,46). In atherosclerosis, increased oxidative stress is associated with the severity of CAD and MI (47,48). Additionally, ROS produced by monocytes can convert oxidized low-density lipoprotein to highly oxidized low-density lipoprotein, which is phagocytosed by macrophages to form foam cells (49).

In summary:

• Inflammation plays a role in the development and progression of AF, atherosclerosis, and cancer.
• IL-1 and the NLRP3 inflammasome are of particular clinical interest, as pharmacologic inhibition of IL-1 was shown in the CANTOS trial to reduce cardiovascular risk, with some signal of decreased cancer mortality as well.
• Inflammation’s role in the development of AF, atherosclerosis, and cancer may be mediated in part by the production of ROS.
• Further studies are needed to delineate the role of anti-inflammatory therapies in cancer prevention or treatment and cardiovascular disease.

## Common Mechanistic Links Between Cancer and AF

AF is the most common atrial arrhythmia, affecting approximately 2% of the general population and with increasing prevalence with age, with 18% of those 85 years and older having AF (50,51). Cancer and AF share similar risk factors, including diabetes mellitus, obesity, and cardiometabolic disease (52, 53, 54). However, recent emerging data suggest that patients with cancer have a higher prevalence of AF compared with the general population (55). One large, Danish nationwide study showed that patients with cancer had an incidence of AF of 17.4 per 1,000 person-years compared with 3.7 per 1,000 person-years in patients without cancer (56). Risk for developing AF after cancer diagnosis was highest in the first 90 days. Another nationwide study of patients with breast cancer showed an increased risk for AF among patients with breast cancer who were <60 years of age compared with the general population (57). Conversely, there is some evidence that AF itself may portend an increased risk for cancer diagnosis (58,59). However, whether this is due to detection bias and not a causal relationship is still up for debate (60). Regardless, AF and cancer share common pathophysiology and risk factors.

Treatment-related factors have been associated with the risk for the development of AF in patients with cancer (61). Postsurgical AF is common in patients with cancer, particularly in those with lung cancer (62, 63, 64). Cancer treatment (chemotherapy, radiation, etc) can also predispose patients to infectious complications, including sepsis, which can promote the development of AF (65). Some traditional cytotoxic chemotherapeutic agents have been associated with the development of AF, including platinum-based chemotherapy, paclitaxel, ifosfamide, corticosteroids, and immunotherapy (66,67). The advent of targeted therapy, including tyrosine kinase inhibitors (TKIs), in cancer treatment has recently changed the way many different types of malignancies are treated. However, these therapies have been implicated in the development of AF in patients with cancer. Ibrutinib is a Bruton TKI that is used for treatment for a variety of B-cell malignancies and is the TKI most associated with an increased risk for AF, with up to 16% of patients diagnosed with AF after initiation of therapy (68, 69, 70). The mechanism behind the development of AF in patients may be due to off-target inhibition of other tyrosine kinases in cardiac myocardial cells (71). For example, ibrutinib has been found to inhibit C-terminal Src kinase. The absence of C-terminal Src kinase in a knockout mouse model was found to induce left atrial enlargement, fibrosis, and inflammation, leading to increased AF (72). Additionally, ibrutinib may also induce AF via production of ROS (73). Immune checkpoint inhibitors are also commonly used in the treatment of certain cancers and have been known to cause cardiotoxicity, myocarditis, and AF driven by dysregulated inflammation (74).

In summary:

• AF is common in patients with cancer, and both diseases share several risk factors, including diabetes and cardiometabolic diseases.
• Treatment-related factors, including traditional chemotherapy and TKI use, can predispose patients with cancer to AF.
• Potential therapeutic strategies to mitigate risk for AF in patients with cancer are yet to be explored.

## Common Links Between Cancer and Atherosclerotic Heart Disease

Cancer and CAD share overlapping risk factors, including but not limited to obesity, smoking, diabetes, and age. Additionally, CAD and cancer may influence the progression of each other. MI has been shown to accelerate breast cancer growth and increase cancer-related mortality (75,76). A nationwide study in South Korea showed an increased risk for new malignancy after percutaneous coronary intervention (PCI) compared with age- and sex-matched control subjects who had not undergone PCI (77). Interestingly, the study showed that the risk for lung and hematologic cancers was higher than that for other types of cancer. Obesity, defined as a body mass index of 30 kg/m^2^ or greater, is a recognized independent risk factor for CAD and atherosclerosis (78). Obesity has also been shown to be a risk factor in the development of cancer, particularly gastrointestinal adenocarcinomas, breast, ovarian, uterine, renal, and liver cancers and multiple myeloma (79). Bariatric surgery for morbid obesity has shown associations with lower rates of both cardiovascular mortality and cancer-related mortality as well as decreased risk for incident cancer (80,81). Diabetes is another classic risk factor for CAD that is also associated with an increased risk for malignancy, especially colorectal cancer. The risk for developing colorectal cancer was increased by 20% to 38% in patients with diabetes compared with those without (82).

Age is a significant risk factor for both cancer and CAD and is also associated with increased somatic mutations in hematopoietic progenitors, leading to clonal hematopoiesis of indeterminate potential (CHIP). These age-related mutations found in genes such as *DNMT3A*, *TET2*, *JAK2*, and *ASXL1*, although they are also found in myeloid neoplasms including myelodysplastic syndromes, acute myeloid leukemia, and myeloproliferative neoplasms, in CHIP, they do not cause alterations in peripheral blood counts (83). CHIP is present in 10% of patients older than 70 years and confers a 10-fold increased risk for developing hematologic malignancy (83). The role of CHIP in cardiovascular disease is an active area of research, with compelling evidence showing an increased risk of cardiovascular death, CAD, and heart failure compared with patients without CHIP (84, 85, 86, 87). The increased risk for CAD and cardiovascular disease in CHIP is mediated by increased inflammation through increased IL-1β and IL-6 signaling, particularly in *TET2*-mutated CHIP (84,88,89). Mutations in *JAK2* are also seen in myeloproliferative neoplasms, which have also been associated with increased rates of arterial thrombotic events, including MI (90, 91, 92). *JAK2* encodes for a protein important for signal transduction for several inflammatory cytokines and hematopoietic growth factors. In a mouse model of atherosclerosis, mice that received bone marrow transplants from *JAK2*-mutated mice had accelerated atherosclerosis and larger plaques with increased necrotic cores (93). Additionally, *JAK2* mutations lead to a thrombophilic state via alterations of platelet and endothelial adhesion molecule activation and increased formation of neutrophil extracellular traps (94,95). Pharmacologic inhibition of *JAK2* in another mouse model of atherosclerosis led to decreased atherosclerotic burden (96). Tumor-associated macrophages and inflammatory cells are important participants in tumor growth and progression, so CHIP may also affect patients with solid malignancies (97). Indeed, mutations associated with CHIP have been shown to be present in solid tumors as well as peripheral blood (98, 99, 100). Additionally, one study showed that 4.5% of patients with solid malignancies harbored mutations associated with CHIP (101). The presence of CHIP was associated with increased age, prior radiation therapy, and tobacco use, and patients with CHIP had shorter survival compared with those without (101).

Although pre-existing factors may affect the risk for developing CAD and cancer, in patients with established cancer, the treatment for their malignancy may also have adverse effects on the genesis and progression of CAD. Radiation, which is used in the treatment of various types of cancers, has been shown to increase the risk for ischemic heart disease (102, 103, 104). In one study, patients who received thoracic radiation for lung cancer had progression in coronary artery calcium, a known predictor of CAD, compared with those who did not (103). Various classes of cytotoxic chemotherapy have been known to cause cardiotoxicity, cardiomyopathy, and vascular toxicity, which may contribute to increased atherosclerotic risk (105). Cisplatin and other platinum-based chemotherapeutics have been associated with an increased risk for MI via endothelial damage and plaque erosion (106). Inhibition of tumor angiogenesis via blockade of vascular endothelial growth factor either by anti–vascular endothelial growth factor monoclonal antibody (bevacizumab) or tyrosine kinase inhibition (sunitinib, pazopanib, and sorafenib) is associated with increased arterial thrombotic events, including MI (107). In a mouse model of atherosclerosis, vascular endothelial growth factor inhibition led to accelerated atherosclerosis and endothelial dysfunction (108). Additionally, exacerbation of hypertension and development of new hypertension are well-known adverse events of vascular endothelial growth factor inhibitors and add to their cardiovascular risk (105).

As targeted and immune-based therapies that have revolutionized cancer therapy are more routinely used, cardiovascular complications of these modalities have been observed. In chronic myeloid leukemia, the identification of *BCR*-*ABL* fusion gene, which leads to overproduction of a growth-stimulatory tyrosine kinase, led to the development of targeted TKIs with imatinib targeting *BCR*-*ABL* as the first. Although the risk for vascular events is relatively low with imatinib therapy, newer generations of TKIs, including dasatinib, nilotinib, and ponatinib, were shown to have substantially higher rates of vascular events compared with imatinib (109). Inhibition of off-target tyrosine kinases, including vascular endothelial growth factor, may explain this increased risk for vascular events (110). Interestingly, not all TKIs may have adverse effects on atherosclerosis. One preclinical study showed that treatment of a mouse model of atherosclerosis with erlotinib, an epidermal growth factor inhibitor, reduced plaque size, likely mediated by decreased T cell plaque infiltration and proliferation (111). Immune checkpoint inhibitors are novel therapeutic agents with expanding use in a variety of different types of cancers but may be associated with increased risk for vascular events (112). Recent evidence suggests that the use of immune checkpoint inhibitors is associated with a 3-fold increased risk for cardiovascular events. Additionally, imaging before and after immune checkpoint inhibitor use showed 3-fold increased progression of total aortic plaque volume (113).

In summary:

• CHIP is a risk factor for both atherosclerosis and hematologic malignancies and may also be associated with worse outcomes in solid tumors.
• Adverse effects of CHIP on cardiovascular outcomes and cancer may be due to inflammation.
• Cancer-related treatments, including radiation, conventional cytotoxic chemotherapy, TKIs, and immunotherapy, can increase cardiovascular risk.

## Role of Platelets and Antiplatelet Therapy in Cancer and Atherosclerotic Heart Disease

**Figure 2:**
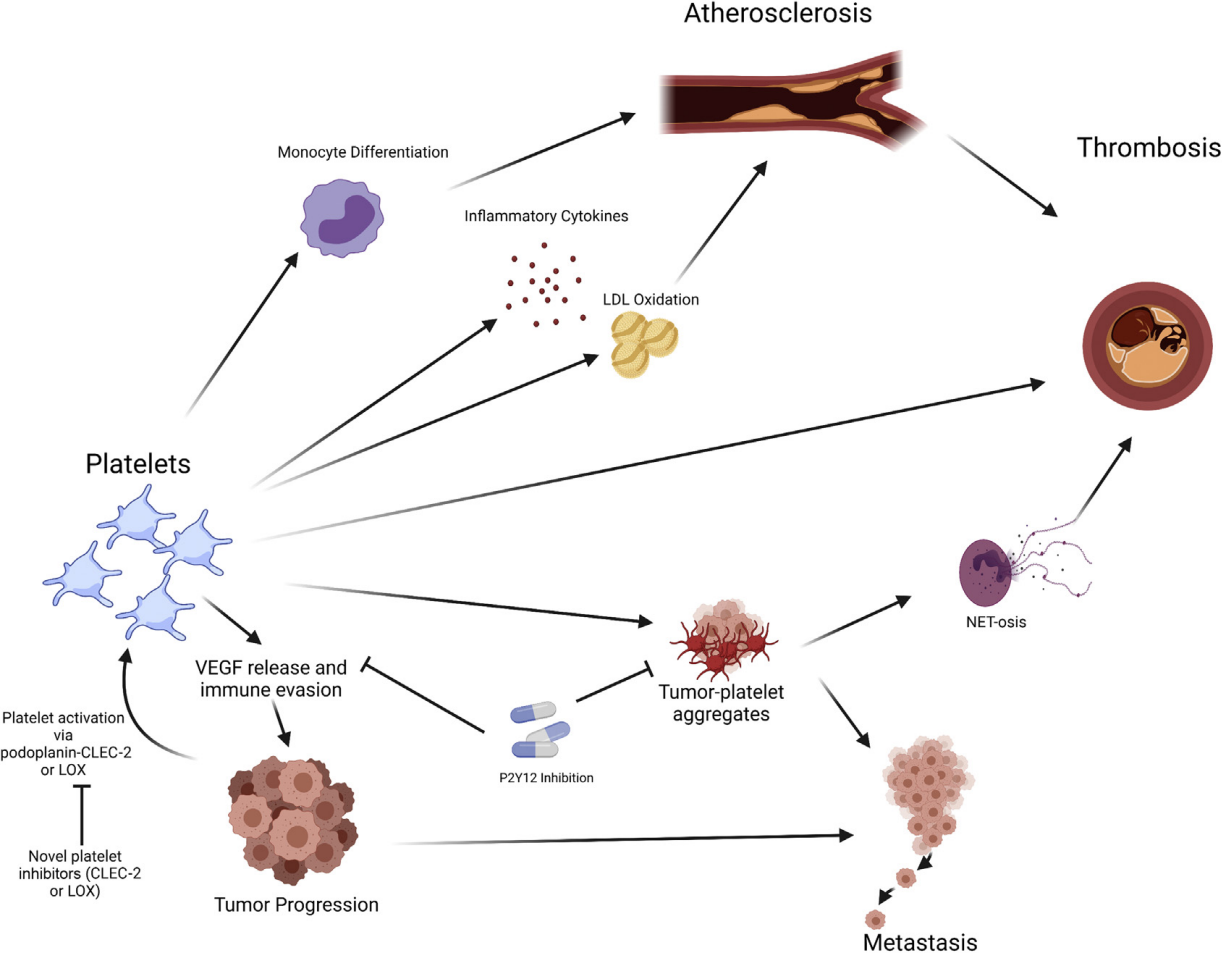
Platelets Are Involved in the Progression of Atherosclerosis and Cancer **Arrows** indicate relationships: platelets facilitate progression of atherosclerosis through monocyte differentiation, release of inflammatory cytokines and low-density lipoprotein (LDL) oxidation. Additionally, platelets contribute to cancer progression and metastasis through release of proangiogenic factors, immune evasion, and hematogenous spread via tumor-platelet aggregates. P2Y_12_ inhibition may attenuate platelet-facilitated tumor progression. CLEC-2 = C-type lectin-like type II transmembrane receptor; LOX = lysyl oxidase; NET = neutrophil extracellular trap.

Platelets are small, anucleate cells with a complex transcriptome and play an important role in hemostasis, inflammation, and immune surveillance (114, 115, 116). Platelets are yet another link between atherosclerosis and cancer (Figure 2) (117, 118, 119). In atherosclerosis, activated platelets contribute to the inflammatory milieu by releasing various platelet-derived mediators of inflammation, including CD40 ligand, IL-1β, platelet-derived growth factor, transforming growth factor-β, ROS, and P-selectin (114,120,121). Platelets have been shown to oxidize low-density lipoprotein, which is a major driver of atherosclerotic plaque formation (122,123). Additionally, platelets also promote monocyte migration to atherosclerotic plaques and induce their differentiation to an inflammatory phenotype, leading to plaque growth and foam cell formation (124,125). Platelets also interact with neutrophils and eosinophils to promote atherosclerotic plaque formation, growth, and thrombosis by inducing neutrophil extracellular trap or eosinophil extracellular trap formation, respectively (126, 127, 128). Increased platelet activation, as measured by urinary levels of 11-dehydro-thromboxane B_2_ (a metabolite of thromboxane A_2_), has also been associated with excess vascular risk in patients with AF despite oral anticoagulation (129).

Platelets have an important role in cancer development, progression, and metastasis (117,119,130,131). Thrombocytosis has been associated with worse outcomes in several types of cancer, including lung, colorectal, ovarian, and hepatocellular carcinoma (132, 133, 134, 135). Additionally, in a mouse model of metastatic lung cancer, induced thrombocytopenia led to significant improvement in survival (136). Tumors may also express proteins that bind to platelets, including podoplanin, which induces platelet aggregation through C-type lectin-like receptor 2 (CLEC-2) and facilitates hematogenous spread and thrombosis (137,138). Additionally, similar to their role in atherosclerosis, platelets facilitate the recruitment of inflammatory cells to the tumor, which contribute to tumor progression (139,140). Hematogenous tumor metastasis is facilitated by platelets by the formation of tumor-platelet thrombi via interactions with tumor cells and leukocytes through P-selectin or tissue factor (141,142). Platelets and tumor cells themselves have been shown to induce the formation of neutrophil extracellular traps, which in turn mediate some of the prothrombotic characteristics of malignancy as well as facilitate metastasis (143). Platelets also facilitate tumor immune evasion through protection from natural killer cells via the transfer of major histocompatibility complex class I from platelets to tumor and T-cell immune regulation (144,145). Platelets have also been shown to express programmed death ligand-1 and help tumors that are programmed death ligand-1 negative evade T cell-mediated immunity (146). Neoangiogenesis, important to tumor growth and metastasis, is enhanced by platelets via release of angiogenic factors, including vascular endothelial growth factor (147). Tumors can also affect platelet behavior by increasing platelet activation, release of microparticles, extracellular vesicles, and platelet granules, which may accelerate atherosclerotic plaque and increase the risk for cardiovascular events (119,148).

Although the benefits of antiplatelet therapy in primary and secondary prevention of CAD are well known (149), there is increasing evidence that antiplatelet therapy may have a benefit on cancer progression (146,150,151). Aspirin is an irreversible antagonist of cyclooxygenase-1 and -2, leading to decreased conversion of arachidonic acid to prostanoids, including thromboxane A_2_ (152). At low doses, aspirin exerts an antiplatelet effect via decreased thromboxane A_2_ production, and at high doses, it provides an anti-inflammatory effect. Aspirin may exert antitumor properties via tumor cyclooxygenase-1 and -2 inhibition and decreased platelet secretion of proangiogenic factors (131). An analysis of 5 aspirin trials showed that daily aspirin therapy was associated with reduced risk for distant metastasis, particularly in patients with adenocarcinoma (153,154). Another large cohort study involving more than 18,000 patients showed that long-term aspirin use of more than 5 years was associated with a decreased incidence of colorectal cancer and prostate cancer in men (155). In addition to reduced risk for metastatic disease, aspirin may reduce the incidence of cancer and risk for cancer-related mortality, though aspirin therapy may have to be longer than 5 years to see the benefit, as other trials with shorter follow-up times have failed to show similar decreases in risk and incidence of cancer (156, 157, 158). Other antiplatelet agents, such as P2Y_12_ inhibitors, have shown some preliminary evidence that they may be beneficial in certain types of malignancies, in part by preventing platelet activation–induced granule release (159,160). In mouse models of hepatocellular carcinoma associated with chronic hepatitis B virus infection and ovarian cancer, treatment with clopidogrel and ticagrelor, respectively, improved survival and tumor burden (160,161). Additionally, P2Y_12_ inhibition appeared to reverse some of the platelet-mediated immune evasion by tumor cells and disrupt tumor cell–induced platelet aggregates, which may be important for hematogenous metastasis (145,151).

Despite the possible antitumor effects of platelet inhibition via aspirin or P2Y_12_ inhibitors, the increased risk for bleeding using these products may limit their clinical usefulness in patients with cancer, thus necessitating the development of antiplatelet agents that do not produce the same bleeding risk. Alternative mechanisms of platelet inhibition that may benefit cancer survival include inhibition of CLEC-2, a platelet receptor that causes platelet activation and binds to podoplanin on cancer cells. In animal models, inhibition of CLEC-2 reduced podoplanin-induced aggregation and hematogenous metastases (137,162). Additionally, inhibition of CLEC-2 did not result in increased bleeding time in mice treated with a podoplanin-CLEC-2 inhibitor (162). Given that podoplanin has also been shown to be expressed in advanced atherosclerotic lesions, and increased levels of soluble CLEC-2 may be associated with increased risk for CAD and may predict death and vascular events in patients with stroke, inhibition of CLEC-2 and podoplanin may be a novel therapy for cardiovascular disease that has yet to be investigated in clinical trials (163, 164, 165). Inhibition of lysyl oxidase, an enzyme more commonly known for crosslink formation of collagen and elastin, provides another novel method of platelet inhibition that may be beneficial in both atherosclerotic disease and cancer. Lysyl oxidase has been shown to be elevated in patients with certain malignancies, including myeloproliferative neoplasms, which are associated with increased thrombotic risk (166). Lysyl oxidase is also a proangiogenic factor that promotes neoangiogenesis in some solid cancers by inducing vascular smooth muscle migration and proliferation (167). In atherosclerotic heart disease, lysyl oxidase has been found to be present in atherosclerotic lesions and to be associated with arterial restenosis after arterial balloon angioplasty (168, 169, 170). In myeloproliferative neoplasms, platelets have been shown to express lysyl oxidase, and increased lysyl oxidase activity is associated with increased platelet adhesion to collagen and increased arterial thrombus formation in a mouse model (166,171). In preclinical studies, inhibition of lysyl oxidase has been shown to attenuate progression of myelofibrosis, a myeloproliferative neoplasm, and triple-negative breast cancer and thus is a promising novel therapy (172, 173, 174).

In summary:

• Platelets are important in the development of both atherosclerosis and cancer via release of proinflammatory and angiogenic factors and facilitating hematogenous spread of cancer cells.
• Platelet inhibition can be beneficial in both atherosclerosis and cancer, but higher bleeding risk may limit the use of traditional platelet inhibitors in patients with cancer.
• The development of novel platelet inhibitors that do not increase bleeding risk, such as inhibition of CLEC-2 or lysyl oxidase, is needed.

## Thrombotic and Bleeding Risk in Patients With Cancer

Patients with cancer are at an increased risk for thrombotic events, both arterial and venous. Tumor-, patient-, and treatment-related factors all play a role in the thrombophilic state of malignancy (6). Venous thromboembolism (VTE) is a well-known and common complication of cancer and cancer therapy, occurring in 20% to 25% of all patients with cancer (175,176). The occurrence of VTE in patients with cancer also portends a worse prognosis, with patients who have had VTE having a 4-fold increase in risk for death compared with patients without VTE (177). Several patient-, treatment-, and tumor-related factors affect the risk for VTE in cancer (6). Additionally, interruption of anticoagulation for procedures or chemotherapy-induced thrombocytopenia has been described to increase thrombotic complications in patients with cancer (178, 179, 180). New risk factors for VTE, including tumor genetics and immune checkpoint inhibitor therapy, are being described and may improve the risk/benefit ratio of pharmacologic thromboprophylaxis (181,182).

The risk for thrombosis in patients with cancer with AF has been studied in retrospective studies. One study of elderly patients with AF found an increased risk for thromboembolic events in patients with lung cancer (183). However, in a Swedish retrospective cohort study of patients with AF off anticoagulation with and without cancer, patients with cancer did not have an increased risk for ischemic stroke after multivariable analysis (adjusted hazard ratio [HR]: 0.91; 95% confidence interval [CI]: 0.88-0.96), though they did have a significantly increased risk for intracranial (adjusted HR: 1.16; 95% CI: 1.05-1.30) and gastrointestinal (adjusted HR: 1.30; 95% CI: 1.22-1.39) hemorrhage (184). The results on gastrointestinal hemorrhage were heterogeneous, with some cancers (colorectal, prostate, myeloma, ovarian, pancreatic) showing significant risk, while others (breast, lung, biliary) did not (184). In another study of patients with cancer undergoing chemotherapy, advanced cancer stage was associated with increased risk for stroke but not chemotherapy itself after adjustment for cancer status (HR: 1.26; 95% CI: 0.78-2.03) (185). Another large retrospective cohort study of more than 2 million adults in France who had been hospitalized with AF investigated the effect of cancer on overall and cardiovascular mortality, ischemic stroke, and bleeding (186). Of the 2,435,541 patients, 399,344 had cancer, and those with cancer had increased all-cause mortality (HR: 2.00; 95% CI: 1.99-2.01) and major bleeding (HR: 1.27; 95% CI: 1.26-1.28) but not ischemic stroke. The risk for bleeding increased with higher HAS-BLED score (C index >0.70). However, the effect of cancer on ischemic stroke was heterogeneous among different cancer types, being elevated in patients with pancreatic, breast, and uterine cancer compared with those without cancer (186). Although the CHA_2_DS_2_-VASc score has been validated in estimating thrombotic risk associated with AF in the general population, it does not include cancer status and may underperform in the cancer population (187). Although the CHA_2_DS_2_-VASc score may underperform in the cancer population, it did predict ischemic stroke in this population, whereas the Khorana score, a VTE risk prediction score for cancer-associated VTE, did not (188). Given the differences in thrombotic and bleeding risks between the cancer and noncancer populations, more comprehensive risk scores need to be developed to adequately predict thrombohemorrhagic complications in patients with cancer.

Cancer is not a single entity, and tumor behavior can differ enormously among cancer types and with different treatment modalities, so it is expected that cancer type and tumor-specific factors can alter thrombotic risk in patients with cancer. Extrapolating from cancer-associated VTE risk research, tumors originating from stomach, pancreas, lung, lymphoma, gynecologic, bladder, and testicular cancer have the highest risk for thrombosis (189). Additionally, tumor genetics likely influence thrombosis risk (181). Patients with anaplastic lymphoma kinase and ROS1 rearranged non-small-cell lung cancer have a 2- to 5-fold increase in thrombosis risk compared with patients without those rearrangements (181,190). The heterogeneity of thrombosis risk among patients with cancer limits the conclusions that can be reached in analyses of randomized controlled trials that enrolled multiple tumor types, although some subanalyses and post hoc analyses that group similar types can yield actionable results (191).

Patients with malignancy may also be at an increased risk for complications following PCI for acute coronary syndrome and other CAD complications compared with the general population, though the data are mixed for thrombotic complications. In a nationwide database of patients who recently underwent PCI, patients with cancer had an increased risk for readmission for acute MI compared with those without cancer (192). The risk for readmission for acute MI was highest among lung and colon cancer (12.1% and 10.8%, respectively) compared with 5.6% for those without cancer. In another large nationwide database study of patients admitted for acute MI, patients with active cancer had a 2-fold increased risk for all-cause death, cardiac complications, and bleeding. Patients with lung cancer had the worst mortality (odds ratio: 2.71; 95% CI: 2.62-2.80) and cardiac complications (odds ratio: 2.38; 95% CI: 2.31-2.45), while patients with colon cancer had the highest rates of bleeding (odds ratio: 2.82; 95% CI: 2.68-2.98) (193). Cancer patients also had increased risk for recurrent acute coronary syndrome or death (15.2% vs 5.3%; *P* < 0.001) in a registry study of patients who underwent PCI for acute coronary syndrome (194). Another registry study from Japan involving 3,499 patients with acute MI treated with PCI showed increased all-cause death in patients with cancer (adjusted HR: 2.43; 95% CI: 1.73-3.42) but no increased incidences of cardiovascular events including acute MI (adjusted HR: 0.71; 95% CI: 0.35-1.42) and stroke (adjusted HR: 1.41; 95% CI: 0.67-2.97) (195). Despite the increased thrombotic risk in patients with cancer, PCI has still shown the benefit of decreased major adverse cardiovascular events in patients with cancer who had acute coronary syndrome if performed within 72 hours of admission and may be underused in this patient population (196, 197, 198). Patients with malignancy are less likely to undergo stenting, receive drug-eluting stents, receive newer generation P2Y_12_ inhibitors (ticagrelor or prasugrel), and receive standard of care medications (beta-blockers, statins, or angiotensin-converting enzyme inhibitors) compared with those without cancer (193,194).

In summary:

• Patients with cancer and AF are at high risk for bleeding.
• Risk for thrombosis in patients with cancer and AF is more heterogeneous, but certain cancer types (pancreatic, breast, uterine) and somatic mutations (anaplastic lymphoma kinase rearrangements in lung cancer, etc) may have an increased risk compared with patients without cancer.
• Risk scores for AF do not include cancer status and may underperform in this patient population, necessitating the development of cancer-specific risk scores in AF.
• Patients with cancer and CAD have increased adverse outcomes and are less likely to receive standard of care compared with those without cancer, presenting an opportunity for physicians to improve post-MI care in this patient population.

## Conclusions and Future Directions

Cancer, AF, and CAD share common mechanistic links and risk factors. Inflammation is a major contributor to the progression of malignancy and cardiovascular disease. Although anti-inflammatory therapies show potential in the treatment and prevention of CAD and may reduce the risk for development of certain cancers, more studies are needed to ascertain the risks and benefits in patients with active malignancy. As cancer treatments advance and prognosis improves, patients with cancer will live longer and be at risk for developing AF and CAD. Compared with those without cancer, patients with cancer and AF or CAD have worse outcomes, including increased thrombotic and bleeding risks. Furthermore, cancer-specific factors including tumor biology and genetics may affect thrombotic risk and more studies are needed to identify high-risk patients. Traditional risk scores underperform in assessing risk for thrombosis and bleeding in patients with cancer, and development of cancer-specific risk scores or inclusion of cancer status in current risk scores may help clinicians navigate these nuanced decisions. Despite the increased risk for recurrent acute coronary syndrome, patients with cancer are less likely to be adequately treated or undergo stenting compared with patients without cancer. The duration of dual-antiplatelet therapy and choice of P2Y_12_ inhibitors in patients are unmet clinical needs and in need of further investigation. Additionally, inhibition of platelets may be beneficial in cancer, as it is in CAD, though bleeding risk limits the routine use of current antiplatelet agents. The development of novel pathways to inhibit platelet activation may prove to be beneficial in this patient population. Further investigation of the common underlying pathophysiology of cancer and cardiovascular disease is warranted and may lead to novel therapeutic and diagnostic strategies to improve the care of this growing patient population (Central Illustration).

**Central Illustration:**
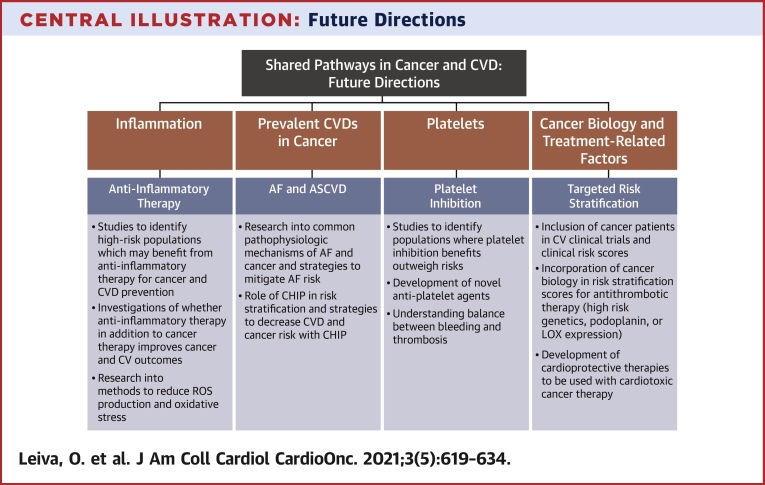
Future Directions Cancer and cardiovascular disease share several common pathophysiologic pathways. Further investigation of underlying mechanisms and development of novel therapeutics and diagnostic strategies may improve the care of this patient population. ASCVD = atherosclerotic cardiovascular disease; CHIP = clonal hematopoiesis of indeterminate potential; CV = cardiovascular; CVD = cardiovascular disease; LOX = lysyl oxidase; ROS reactive oxygen species.

## Funding Support and Author Disclosures

Dr Bhatt is an advisory board member for Cardax, CellProthera, Cereno Scientific, Elsevier Practice Update Cardiology, Janssen, Level Ex, Medscape Cardiology, MyoKardia, Novo Nordisk, PhaseBio, PLx Pharma, and Regado Biosciences; is on the boards of directors of the Boston VA Research Institute, the Society of Cardiovascular Patient Care, and TobeSoft; is chair of the American Heart Association Quality Oversight Committee; is a member of data monitoring committees for the Baim Institute for Clinical Research (formerly Harvard Clinical Research Institute, for the PORTICO trial, funded by St. Jude Medical, now Abbott), the Cleveland Clinic (including for the ExCEED trial, funded by Edwards Lifesciences), Contego Medical (chair, PERFORMANCE 2), the Duke Clinical Research Institute, the Mayo Clinic, Mount Sinai School of Medicine (for the ENVISAGE trial, funded by Daiichi Sankyo), and the Population Health Research Institute; has received honoraria from the American College of Cardiology (senior associate editor, *Clinical Trials and News*, ACC.org; vice chair, ACC Accreditation Committee), the Baim Institute for Clinical Research (formerly Harvard Clinical Research Institute; RE-DUAL PCI clinical trial steering committee funded by Boehringer Ingelheim; AEGIS-II executive committee funded by CSL Behring), Belvoir Publications (editor-in-chief, *Harvard Heart Letter*), the Canadian Medical and Surgical Knowledge Translation Research Group (clinical trial steering committees), the Duke Clinical Research Institute (clinical trial steering committees, including for the PRONOUNCE trial, funded by Ferring Pharmaceuticals), HMP Global (editor-in-chief, *Journal of Invasive Cardiology*), the *Journal of the American College of Cardiology* (guest editor, associate editor), K2P (cochair, interdisciplinary curriculum), Level Ex, Medtelligence/ReachMD (continuing medical education steering committees), MJH Life Sciences, the Population Health Research Institute (for the COMPASS operations committee, publications committee, steering committee, and US national coleader, funded by Bayer), Slack Publications (chief medical editor, *Cardiology Today’s Intervention*), the Society of Cardiovascular Patient Care (secretary/treasurer), and WebMD (continuing medical education steering committees); is deputy editor of *Clinical Cardiology*; is chair of the NCDR-ACTION Registry Steering Committee and the VA CART Research and Publications Committee; has received research funding from Abbott, Afimmune, Amarin, Amgen, AstraZeneca, Bayer, Boehringer Ingelheim, Bristol Myers Squibb, Cardax, Chiesi, CSL Behring, Eisai, Ethicon, Ferring Pharmaceuticals, Forest Laboratories, Fractyl, HLS Therapeutics, Idorsia, Ironwood, Ischemix, Janssen, Lexicon, Lilly, Medtronic, MyoKardia, Novo Nordisk, Owkin, Pfizer, PhaseBio, PLx Pharma, Regeneron, Roche, Sanofi, Synaptic, and The Medicines Company; has received royalties from Elsevier (editor, *Cardiovascular Intervention: A Companion to Braunwald’s Heart Disease*); is a site coinvestigator for Abbott, Biotronik, Boston Scientific, CSI, St. Jude Medical (now Abbott), and Svelte; is a trustee of the American College of Cardiology; and has conducted unfunded research for FlowCo, Merck, and Takeda. All other authors have reported that they have no relationships relevant to the contents of this paper to disclose.
